# Physic for Uterine Disease

**Published:** 1877-06

**Authors:** 


### Editorial.

### PHYSIC FOR UTERINE DISEASE.

Most physicians, and *all* their patients, have been disappointed and disgusted with the result of the iron, assafœtida, opium, bromide potassium, and digestive tonic treatment used for months and years in chronic uterine disease. A practitioner who has proved the uselessness of this everlasting medication constitutionally, as it is termed, cannot conscientiously continue the practice. He well knows the fees obtained for prescriptions made without any hope of permanent benefit, where such benefit is expected by the patient and friends, are not just, strictly speaking. We may quiet our conscience by the reflection that we have done the best *we can* for one who requests and insists upon our services, but for the conscience of those who know there are local means, if properly applied, that will arrest the disease, this is not sufficient. If permanent relief can be afforded, it is our duty to give the patient the benefit of it. If we have not sufficient mental energy to make ourselves acquainted with the plans of successful treatment, or the physical industry to carry them out, we certainly act unjustly toward those who confide in our honor and faithfulness. Our physical inability to undergo the labor of such treatment, along with the arduous duties of general practice, is not valid excuse for drugging for years the unfortunate subject of such disease. There are those who will give them the necessary treatment, and by retaining them under our charge when it is not intended to give them the proper treatment, we not only exact money for useless service, but become responsible for the continuance of a disease which becomes more and more difficult of cure in proportion to the length of time it exists.

This, to our mind, is a sad picture; but our grief is greatly intensified by the course a few of our medical brethren think proper to pursue. They not only refuse practical familiarity with any plan proposed for curative treatment, but ridicule the idea of such plan to their patient, in order to change any inclination she may have to seek relief by rational means.

Moreover, we have been pained recently by the perusal of a usually well conducted medical journal, in which, editorially, a series of articles appears, evidently intended to discourage and ridicule those whose labors have been constantly, for many years, directed to the relief of suffering females. It is certainly praiseworthy in a journal to attack any unjustifiable and injurious plan of treating disease, and particularly one fraught with so much delicacy as that under consideration, but to pronounce against the merits of an instrument or course of management, which has been the subject of laborious study and practical test by a man of scientific attainments and profound judgment, and that without any experience in the matter, is to use journalism against this sacred cause of humanity. We do not wish to be understood as impugning the motives of the editors, but the *Louisville Weekly News* has certainly struck a blow which, if heeded, will retard, to that extent, the progress being made in gynæcological science. The stem pessary to which the *News* alludes, may or may not be an important addition to the means of curing uterine disease. It is impossible, however, for the writer to have been entirely satisfied on this point, without testing, or, at any rate, without a more thorough study of the subject than he had time to have, after first reading a description of the instrument. We confess to being prejudiced against pessaries of all kinds, believing that many of them increase the difficulty they are intended to relieve, but should by no means condemn an invention of this kind without proper test of its benefits; and particularly would we hesitate to attempt by ridicule to paralyze the influence of those whose labors and advancement exceed our own, in this department of the healing art.

Every truly scientific man who devotes himself to the study of female diseases, should be encouraged by the press and the people in every proper way, instead of retarding his progress by slurs, innuendoes, and ridicule.

